# 3D limb dynamics of flyball dogs turning on different box angles

**DOI:** 10.1038/s41598-024-78863-9

**Published:** 2024-11-13

**Authors:** Scott Blake, Roberta Blake

**Affiliations:** 1 Hartpury University, Hartpury, Gloucester, GL19 3BE UK; 2 Anglia Ruskin University, Lordship Road, Chelmsford, CM1 3RR UK

**Keywords:** Biomechanics, Canine, Sport dogs, Animal physiology, Physiology

## Abstract

There are no regulations for the flyball box angulation, which ranges from 45° to 89°. As such at present, the box turn is deemed to represent the greatest injury risk to competitors. The aim of this study was to understand the influence of box angle on kinematic variables during a flyball turn, by comparing dogs turning on three different angulations of flyball box (45°, 60° and 83°) to allow for recommendations to be made regarding the most appropriate box design in terms of limiting risk of injury across the sport, to increase both wellbeing and safety for competitors. Turning on a 45° box generates significantly more flexion in the forelimbs and carpus, whereas turning on an 83° box generates greater degrees of extension in the elbow, shoulder, hock and stifle. What our 3D analysis has shown is that the relationship between box angle and the physical demands placed on the dog are complex, and related mainly to asymmetrical nature of the sport, and as such no one angle may be more or less suitable for training and competition, but the 60° seems to be a mid-ground, whereas direction of turn may be fundamental in generating the potential for injury.

## Introduction

**Fig. 1:**
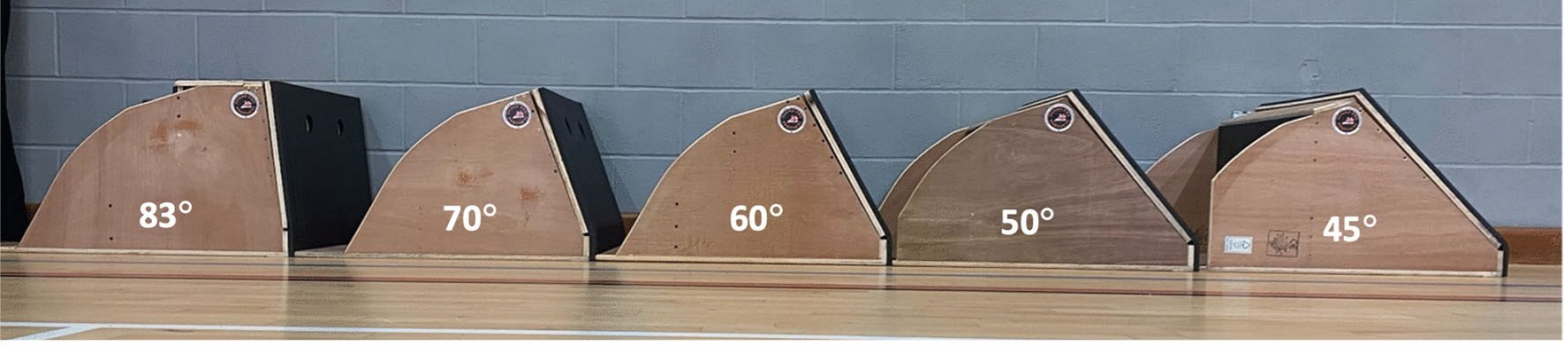
Five different angulations of flyball box.

Flyball is a fast-paced sprint canine sport run in a relay fashion. A variable in flyball is that the angulated surface of the flyball box that releases a tennis ball and which the dog uses to initiate its turn is not standardised, and can range from 45° to 89° (see Fig. 1). Multiple studies have investigated biomechanics of agility and working trial obstacles in canines^1–12^ but have mainly examined the sagittal plane, whereas a box turn requires movement in multiple planes. To date, there is no published research reporting the biomechanical demands of turning in flyball turn, and there is no evidence to understand whether the angulation of flyball box influences biomechanics and consequently the high rate of injury occurrence within the sport^13–16^.

A 180° change of direction at speed to negotiate the flyball box requires the dog to judge its flight arc in relation to the box surface, reduce its velocity to zero whilst retrieving a ball, and generate propulsive forces through the hindlimbs whilst rotating its body in readiness to land squarely, in preparation to re-accelerate back to the start/finish line.

A perfect turn, also known as a swimmer’s turn, is regarded as when a dog makes box contact with all four limbs, with the torso at an angle of approximately 45° behind the vertical. This allows for a fast turn similar to that seen in human swimming, potentially avoiding injury from contacting the box head-on. It could be hypothesised that angles closer to the vertical ensure that a dog slows down prior to take-off, reducing impact forces and giving time to perform an optimum turn, but this can only be confirmed via experimentation. Understanding the amount of flexion and extension of the major joints of the limbs during a flyball turn would allow us to define the risks of injury, but more importantly formulate a hypothesis regards methods of reducing harm and improving welfare for canine participants.

The aim of this study was to understand the influence of box angle and direction of turn (differences between inner and outer limb on the box turn) on 3d kinematic variables during a flyball turn, by comparing dogs turning on three different angulations of flyball box (45°, 60° and 83°). We hypothesised that biomechanics demands will be higher on the contact with boxes that are closer to 90° than 45°.

## Results

A video of one flyball box turn can be seen in *Supplementary video 1* and one 3d video analysis of a flyball turn can be seen in *Supplementary video 2.*

An overview of the main effects and interactions of box angle’, ‘obstacle’ and ‘limb’, as well as the covariance caused by the random effect is shown in Table 1.

**Table 1:** Main effects and interactions of final reduced linear mixed models.

	Carpal Extension	Carpal Flexion	Elbow Extension	Elbow Flexion	Shoulder Extension	Shoulder Flexion	Hock Extension	Hock Flexion	Stifle Extension	Stifle Flexion	Hip Extension	Hip Flexion
*Random Effect*												
Dog	50.2%	69.6%	20.3%	41.9%	13.3%	24.9%	18.1%	25.8%	19.5%	26.4%	12.7%	19.6%
*Fixed Effect*s												
Obstacle A:TO:C:PO:L	p < 0.001*	NA	p < 0.001*	p < 0.001*	p < 0.001*	p < 0.001*	p < 0.001*	p < 0.001*	p < 0.001*	p < 0.001*	p = 0.012*	p = 0.022*
Box angle 45:60:83	p = 0.189	p < 0.001	p = 0.01*	p = 0.002*	p < 0.001*	p = 0.02*	p = 0.005*	p = 0.558	p < 0.001*	p < 0.001*	p = 0.732	p = 0.607
Limb Ld:T/I:O	p < 0.001*	p = 0.609	p = 0.148	p = 0.019*	p < 0.001*	p = 0.709	p < 0.001*	p < 0.001*	p = 0.162	p = 0.637	p < 0.001*	p = 0.281
*Interactions Effects*												
Box angle*Obstacle	p < 0.001	NA	p = 0.02	p = 0.011	p < 0.001	p < 0.001	p = 0.017	p = 0.355	p = 0.209	p = 0.025	p = 0.142	p = 0.092
Box angle*Limb	p < 0.001	p < 0.001	p = 0.185	p = 0.311	p = 0.024	p = 0.696	p = 0.307	p < 0.001	p = 0.019	p = 0.045	p = 0.610	p = 0.428
Obstacle*Limb	p < 0.001	NA	p = 0.221	p = 0.014	p = 0.002	p = 0.028	p = 0.006	p = 0.014	p = 0.021	p < 0.001	p = 0.014	p = 0.528
Obstacle*Limb*Box angle	p < 0.001	NA	p = 0.627	p = 0.002	p = 0.167	p = 0.100	p = 0.233	p = 0.142	p = 0.235	p = 0.422	p = 0.031	p = 0.023

Random effect (Dog) values are shown as individual variance (%). P > 0.05 fields indicate the effect was not included in the final model. The final reduced model for each parameter can be obtained from the non-empty fields in the corresponding table column. For example, the final reduced model for shoulder flexion contains obstacle and box angle as main effects, box angle*obstacle and obstacle*limb interaction as well as the random effect dog. *Interaction-driven main effects.

### Main effects of box angle, obstacle phase and limb

For elbow flexion, shoulder extension and hock extension, all main effects were significant (Table 1). Phase of obstacle had a main effect on all variables. Box angles had a main effect report in most joint angles, except carpal extension, hock flexion and hip flexion and extension. Limbs (whether lead or trail/inner or outer) was the main effect with the least number of significant variables. Most of the main effects were interaction driven and are reported with asterisks on Table 1. Although, we acknowledge that interaction driven main effects do not necessarily need to be reported, we have opted to describe them for clarity.

### Box angle main effect

Across all obstacle phases and limbs, the 45 degrees angle box has elicited more flexion in the carpus (p < 0.001), shoulder (p = 0.02), stifle (p < 0.001), and elbow (p = 0.002). Whilst the 83° box caused more significant increases in joints extension on elbow (p = 0.01), shoulder (p < 0.001), hock (p = 0.005), and stifle (p < 0.001).

### Obstacle phase main effect

Comparing approach, take-off, contact, push-off and landing, the contact phase had the largest number of significant findings, mainly on forelimbs, with higher carpal (p < 0.001), elbow (p < 0.001) and shoulder (p < 0.001) extension, and higher flexion of the shoulder (p < 0.001), elbow (p < 0.001) and hock (p < 0.001) amongst the phases. The push-off phase has shown the most significant effects on hindlimb extension, with the highest extension on hock (p < 0.001), stifle (p < 0.001) and hip (p = 0.012), amongst the phases of the obstacle. Hindlimb flexion overall was higher during approach with hock (p < 0.001), stifle (p < 0.001) and hip (p = 0.022) more flexed during this phase. Landing phase only elicited a higher shoulder flexion (p < 0.001) along with contact.

### Limb main effect

In the forelimbs, carpal was more extended (p < 0.001) on inner limb, whilst shoulder joint was more extended on the outer limb during the contact and turn, and elbow joint was more flexed (p = 0.019) in the trailing limb compared with the lead limb. For hindlimbs, hock and hip joints were more extended on the lead limbs (p < 0.001), whilst hock was more flexed on the trailing limb compared with lead limb (p < 0.001).

### Interactions of box angle, obstacle phase and limb

The interaction term in a linear mixed model (LMM) allows for the possibility to study the interaction of two factors. If the p-value for the interaction term is greater than 0.05, then the interaction is not significant (Table 1).

### Box angle*obstacle phase

On the 45° and 60° boxes, the elbow was more extended on contact than take-off and push-off, respectively (p = 0.02). It was also more flexed on contact than take-off for boxes 45° and 60° (p = 0.011). On both 60° and 83° boxes, carpal joint was more extended whilst in the contact phase (p < 0.001). On the 45° box the shoulder was less extended on take-off than other phases (p < 0.001), but it was more flexed on contact and landing (p < 0.001). On the 60° box, the shoulder was also more flexed on the contact and landing phases. Hock was more extended on approach and take-off on the 60° and 83° boxes, whilst only on approach on the 45° box (p = 0.017). Lastly, the 45° and 60° boxes showed a higher stifle flexion on both approach and contact (p = 0.025).

### Box angle*limb

We found increased values in the trail versus lead forelimbs for carpal extension (p < 0.001) and stifle flexion on the 45° box (p = 0.045); hock flexion (p < 0.001) and shoulder extension (p = 0.024) on the 60° box; and as well as greater shoulder extension on the 83° box. Compared with 45° and 60°, stifle flexion (p = 0.045), carpal flexion (p < 0.001) and shoulder extension (p = 0.024) were higher in the lead hindlimb on the 83° box.

### Obstacle phase*limb

The inner carpus (p < 0.001) and both shoulders (p = 0.002) were more extended on contact when compared with the other phases. The trailing hock was less extended on take-off (p = 0.006) Lead/outer elbow was more flexed on contact than TO (p = 0.014), whilst lead/outer shoulder (p = 0.028) was more flexed on contact and landing.

We found that the lead/outer hock flexed (p = 0.014) more on approach and contact than other phases whilst the trailing hock extends more (p = 0.006) on push off and TO compared to other phases. Conversely, the trailing stifle was more extended (p = 0.021) on contact and approach.

### Individual kinematics variables

Analysis of variance (repeated measures ANOVA or Friedman’s test) was applied to understand better the individual kinematics variables across box angles. The data which presented a statistically significant difference between box angles can be found on supplementary *Tables S1-S6.* A common element found is the 60° box showing more consistently less maximum flexion or extension during the turn*.*

## Discussion

**Fig. 2:**
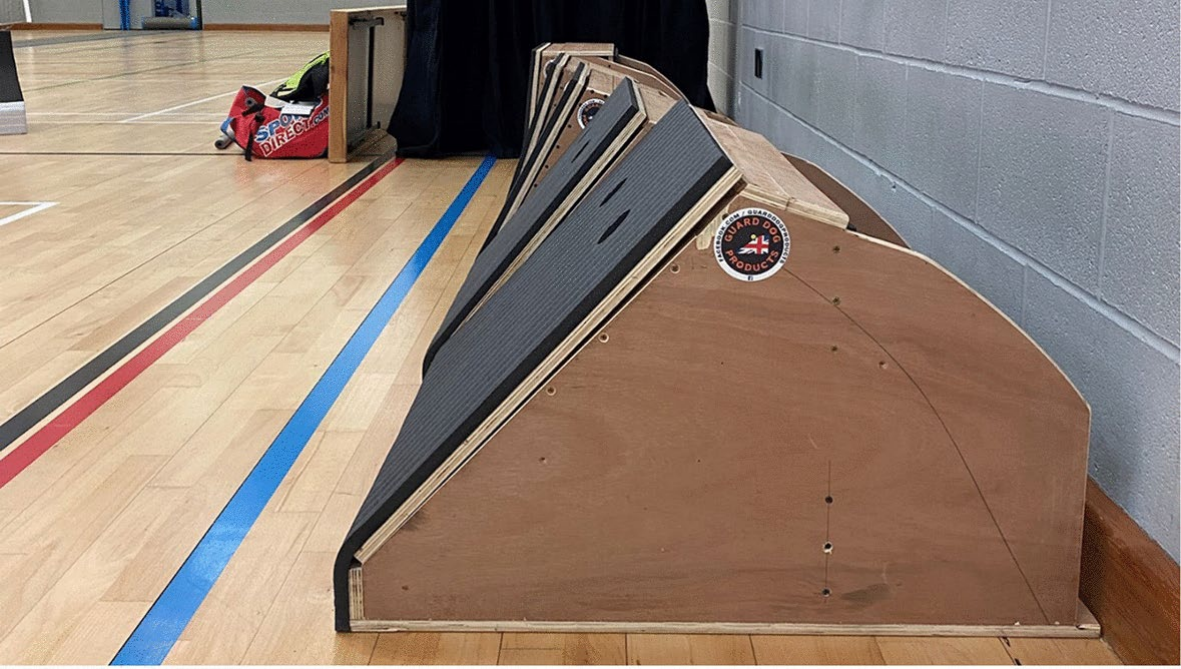
Five flyball boxes angles to illustrate relative distance dog has to reach over the box to collect the ball between 83° (background) versus 45° (foreground).

Our results suggest box angle has some effects in a way a dog completes a box turn but has highlighted significant differences between inner forelimb and outer hindlimb. Some of the results initially seemed counter intuitive, until we understand that the initial position of the ball, its releaseand the dog’s retrieval, all influence kinematic variables in the early part of the turn. If a dog turns left, the ball will be released approximately above and in front of the right forelimb, and if turning right, above and in front of the left forelimb. However, with a box angle of 45° the dog must lean further over the box to be able to able to retrieve the ball, than either a 60 or 83° angle (Fig. 2). The dog is therefore having to reach approximately 52 cm further on a 45° box than an 83° which requires compensatory changes in contact, some of which are described below. This may also account for why dogs are more likely to contact 45° boxes head-on, with forelimbs extended and less rotation of the trunk, which allows them to reach further.

The LMM identified a main effect in hindlimb flexion of the hock, stifle and hip during approach, which is unsurprising considering the dogs need to generate elastic energy in the hindlimbs to assist with take-off^17^ . Furthermore, the greatest amount of flexion was in the lead/outer hock, which, when viewed alongside take-off, suggests this is as a consequence of the trail limb needing to extend to generate rotation for the swimmers turn. When we tested for variance, we could find no evidence of approach kinematics changing depending on box angle, or any variation between inner and outer limb. This would suggest that each dog has a standard approach technique regardless of the box that is presented to them, which is in line with our previous results which showed a low degree of intra-dog variability on approach to the box.

**Fig. 3:**
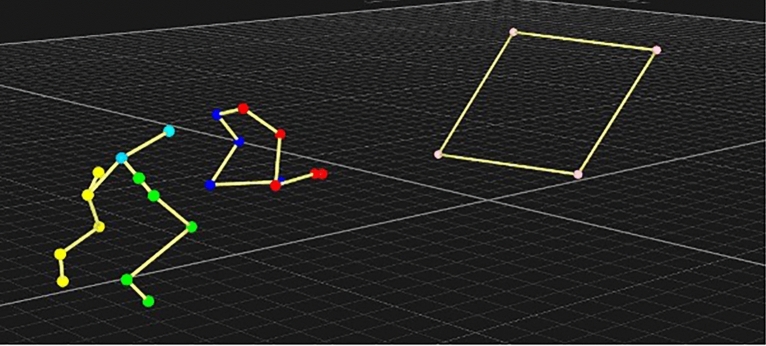
Dog at take off towards 45° box angle.

Our linear mixed model did not find any significant main effects during take-off, but both the LMM and the analysis of variance found that the greatest amount of extension is generated in the trail hock and is greater during take-off towards the 45° and 60° box angles (Fig. 3).

At the same time the contralateral stifle showed significant degrees of flexion, which indicates that vertical force is being created at the hock to gain the necessary height at take-off^18^, whilst at the same time preparing for rotation of the trunk to affect a swimmers turn. An interesting result is that during take-off towards the 45° box, the lead hip has a higher amount of extension, but the shoulder has the least amount when compared to the 83° angulation. We now hypothesise that this is due to the ball being further away and effectively lower at 45° than 83°. This means the dog has to travel further during take-off, to effectively reach over the 45° angle, but at the same time limit extension of the lead shoulder and elbow to be able to lower its head and reach the ball, whereas at 60° and 83° the ball is effectively closer and therefore easier to reach. Stifle extension on the inner limb was lower during take-off towards the 45° angle, with greater extension on the outer limb. Taking ball distance into account would also explain why stifle extension is greater on the outer hind at 45° as due to the slight rotation needed to achieve a swimmers turn, the outside limb has to travel further to contact the box surface, which gets closer as the box angulation increases towards 83°.

**Fig. 4:**
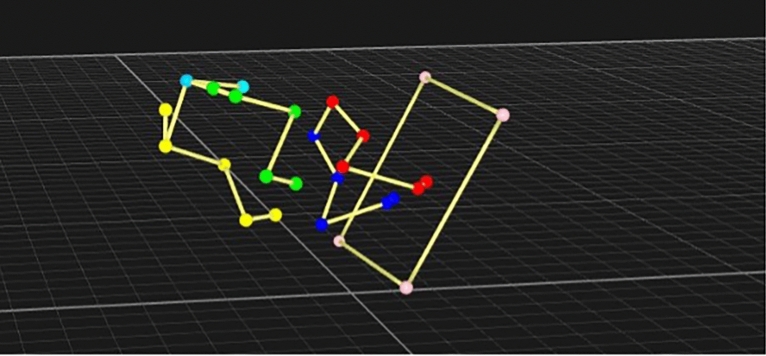
Dog at point of contact with a 60° flyball box.

The LMM identified that box contact had the most significant effect regarding the forelimbs with higher carpal, elbow and shoulder extension, but conversely higher flexion of the shoulder and elbow. When analysis of inner/trail, outer/lead are taken into account, we see that elbow flexion is greater at the inner/trail, most notably at 45° and 60°, which would be in preparation for completing the turn, but carpal and shoulder extension is high in both forelimbs, as both contact the box at approximately the same time. An example of the 3D data for a 60° box contact can be seen in Fig. 4.

**Fig. 5:**
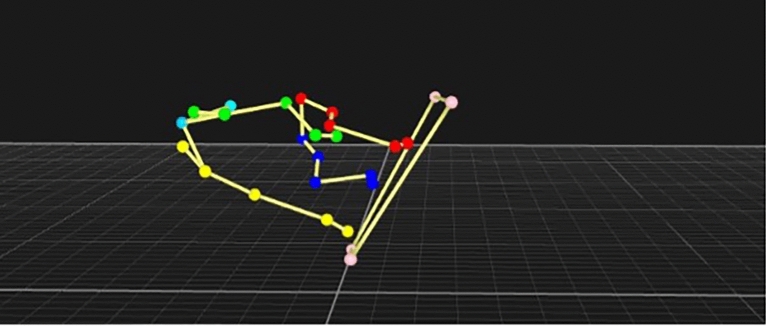
Rearview of a dog at point of contact with a flyball box, with inner hindlimb (yellow) making contact prior to outer hindlimb (green).

Extension of the inner carpal was greater overall, which would be expected considering the need to turn, and shoulder flexion was also greatest across all angles, which is explained by the need to dip the shoulder to be able to retrieve the ball. Our variance analysis also showed that extension of both the stifle and hip was greater on the limb situated on the outside of the box turn, whilst flexion was correspondingly lower on the inner limb, which would also indicate that at contact the dog is partially completing the turn, by flexing its inner limbs to be able easily make ground contact after push-off. This also suggest that rotation of the trunk to meet the box is not complete at contact, because as both forelimbs contact the box, so too does the inner hind limb, whilst inertia of the hind trunk then brings the outer hind into full contact (Fig. 5).

Results also indicate that flexion of the outer hip is significantly greater at 45° than 83°, which we believe is due to the rotation created from the swimmers turn needing to be absorbed, with earlier contact at 45° generating a larger amount of rotation within the trunk. We also found that abduction of the hips is greater at 45° than 83°, which meets our hypothesis regarding distance travelled by the limbs being greater at lower angles. At 45° the hind limbs effectively have to reach further to touch the box, creating greater abduction at the hip. What needs to be considered however is that the abduction will not be entirely symmetrical initially, based on our observations that the inner hind limb will contact the box first as inertia drives the trunk and outer hindlimb towards contact. In the forelimbs we expected to see greater extension of the shoulder and elbow on the outside of the turn, simply due to landing on an angled surface where the base is closer than the peak, however these were not reflected in our results. Extension of the inner carpal was greater overall, which would be expected considering the need to turn, and shoulder flexion was also greatest across all angles, which is explained by the need to dip the shoulder to be able to retrieve the ball.

**Fig. 6:**
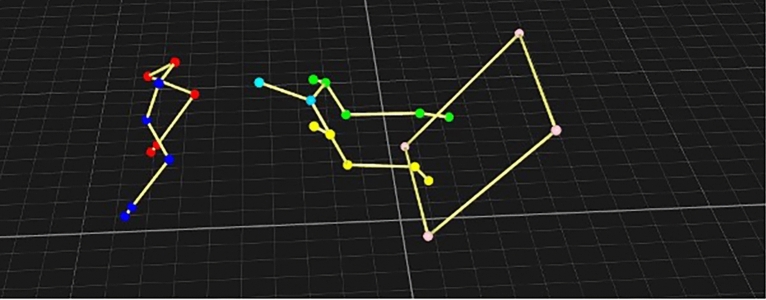
Dog pushing away from 45° flyball box.

Our initial assumption from the data collected in section one was that a primary injury factor within the outside hindlimb was due to the dog needing to push away from the box to complete its turn. The LMM does indicate that this phase generates significant effects on hindlimb extension, generating the highest extension at the hock, stifle and hip (see Fig. 6). Additionally, our variance analysis identified that both hip and hock extension is greater on the outside limb, when compared to the inner, but is less at 45° when compared to the other angles. Stifle extension on the inner limb was also lowest at 45° demonstrating less need to forcefully push away from the box horizontally, which is needed to rotate the trunk back to vertical at higher angles. This may also be a reason why dogs using higher angles are faster overall, as they have more opportunity to forcefully push away from the box and generate momentum as they return to the start/finish line. Observation of both the 3D and 2D video shows that dogs effectively drop the forelimbs away from the box as they turn, but generate landing distance by extending the inner stifle, whilst relying on the outer hindlimbs to generate more vertical motion away from the box. Unsurprisingly, no significant results were identified in the forelimbs during box push-off, as they will be extending in preparation for landing during this period.

**Fig. 7:**
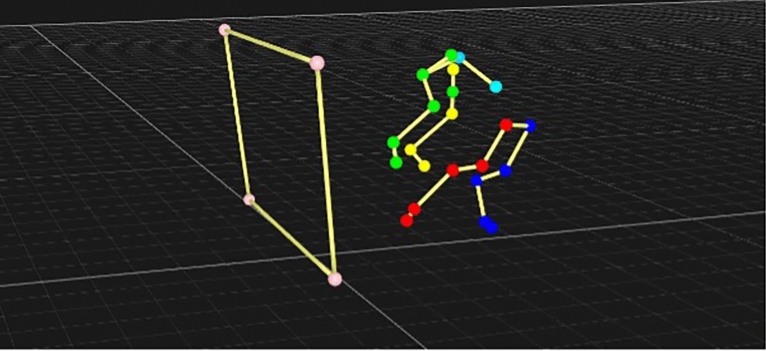
Dog at point of landing from an 83° flyball box.

As previously described, observations show that dogs tend to drop away from the box with the forelimbs as they complete their turn, and as such the inner forelimb acts as trail during recovery as it is closest to the box, which is supported by our results showing that carpal extension was greatest during landing on the inside of the box turn (trail limb), as well as both shoulder and elbow flexion (Fig. 7).

Extension of the carpal was higher than has previously been seen in jumping or A-frame negotiation^9,19,20^, whilst shoulder and elbow flexion was less than previous studies^10^ which would be as a result of the reduced distance to the ground. Shoulder flexion was greatest in the lead limb, which would be as a consequence of the trail forelimb acting as a strut as the CoM passes over it^21^. As the dog first lands the trail limb will carry all of the body mass for a short period of time^22^ but remain relatively extended, so we would expect to see higher peak vertical ground reaction forces during our kinetics investigations. Considering the greatest degree of extension is to the inner carpus on both box contact and landing, it is perhaps surprising that there are relatively few carpal injuries within the sport. We only found one difference between box angles on the forelimbs, where elbow flexion was greater at 60 versus 45, which suggests that box angle does not appear to greatly influence the degree of flexion or extension of the forelimbs upon landing. This supports the hypothesis that dogs have a turn technique which is independent of the angle of the box, however, as previously described, a consistent turn direction will mean that there will be constant, ongoing asymmetry in flexion and extension between the inner and outer limbs.

Although it is not completely clear which box angle could be deemed the safer, it is clear that the turn direction has a great effect in repeated asymmetry. Where the box turn differs from any other obstacle in canine sports is that flyball competitors will only ever turn in one direction, subjecting limbs to repeated, asymmetrical loading, which would go some way to explain the association between turn direction and injury location seen in surveys^15,16^. A similar phenomenon is found in racing greyhounds which mirrors the association identified between injuries at the hip and turn direction. Previous surveys^15,16^, have established that the turn direction does not represent a risk of injury, it does determine where injuries may be more frequent (inner forelimb and outer hindlimb) due to the asymmetric nature of the turn. The shoulder, is more affected on the inside forelimb of the turn in flyball, and it is along hip and groin injuries on the outside limb the most reported injury in flyball dogs^15^. Consistently turning in one direction will therefore subject the dog to repetitive, unbalanced stresses alongside extremes of joint angulation, which will have negative consequences for their musculoskeletal health. The asymmetrical nature of turn movement has been described in other canine sports as in agility wrap jump^21^, Greyhound racing^23^ and in equine sports^24,25^, amongst other species.

As any research, ours had limitations. Due to the difficulty to source a higher number of dogs able to safely perform the three box angles, our sample was limited. A larger sample, with more breeds addressed would make the results more generalisable to the flyball population. In the future, a longer study allowing a lengthy adaptation of a larger sample to all angles would be warranted.

What is clear is that further research is needed to ascertain the extent of biomechanical changes occurring at the box turn, which would need to include an understand of the angle of impact with the box, as well as the forces present at contact, push off and landing. These data could support the making of recommendations regarding training and equipment to improve competitor welfare or limit the types of injury described. Thought should be given by the flyball community of ways to train or encourage dogs to turn in either direction at will or on command. This may lead to a reduction in performance initially until the dog becomes habituated to the new challenge but will be advantageous in the longer-term regarding time off from the sport due to injury, as well as a possible improvement in the dog’s wellbeing.

Our investigation has identified fundamental differences in the phases of turning in flyball dogs. Furthermore, we now know that turning on a 45° box generates significantly more flexion in the forelimbs and carpus, whereas turning on an 83° box generates greater degrees of extension in the elbow, shoulder, hock and stifle. Our 3D analysis has shown is that the relationship between box angle and the physical demands placed on the dog are complex, and as such we cannot definitely answer the question “Is there a perfect angle to prevent sport injuries in flyball dogs?” as no one angle may be more or less suitable for training and competition, but the 60° angle seems to present a good compromise, although further studies are needed to confirm this. In other hand, the direction of turn may be fundamental in generating the potential for injury.

## Methods

The trial was a repeated measure, crossover design.

### Ethical approval

The data was acquired according to modern ethical standards and according to guidelines set by The Animal (Scientific Procedures) Act 1986. The research has been approved by the Hartpury University Ethics Committee, approval number ETHICS2021-54. A written informed consent was also obtained from the owners of the participants of the study prior to data collection. The reporting of the trial has complied with the ARRIVE guidelines.

### Study population

Dogs were recruited from British Flyball Association members who regularly train and compete in flyball in the UK. To be included in the study, dogs had to be over 2 years old, have been regularly training or competing in flyball competitions in the previous 12 months, be physically fit enough to take part in the research project, and free from injury. Each dog had also competed in flyball with one of the flyball boxes angulations used in the study (45°, 60°, and 83°). Furthermore, each dog was deemed by their experienced handler to be sufficiently skilled to be able to complete multiple, repeated flyball runs utilising the three different angulations of flyball box. Sample size was five as very few dogs have the capacity to be able to turn on a variety of different flyball box angles within the course of one data collection. The sample size was however deemed sufficient using the resource equation approach (minimum 5 and maximum 10 dogs)^26^ and has been deemed sufficient in similar published studies assessing canine biomechanics^8^ on obstacles which achieved adequate statistical discrimination with a range of 5–10 dogs. Assuming the methods differ slightly in their intervention and outcomes, estimates by 10%, for Type I and II errors of 0.05 and 0.20, respectively, using the Bland–Altman Test, we estimated a sample size of between 5 to 12 dogs was needed (MedCalc® Statistical Software version 20.115), however only five could be found with the necessary skills required. Handlers confirmed that dogs were free from injury and disease and were examined by an experienced, qualified veterinary physiotherapist prior to data collection to confirm the absence of any musculoskeletal issues or lameness. Four of the dogs were whippet cross breeds, whilst the fifth was a Springer Spaniel. Further demographic data for each participant can be seen in *supplementary table S7.*

### Experimental set-up

A standard flyball course was created in an indoor sports court, consisting of a 4ft x 51ft run, with n = 4 hurdles positioned at 6,16,26 and 36 feet from the start/finish line (BFA,2023). Hurdle height was set at 12 inches, which is the minimum height a dog is expected to jump during a BFA sanctioned competition. A running surface, constructed from 10 mm Tuffspun™ matting commonly used in both training and competition was used to denote the flyball lane. Three standard flyball boxes, each with a different angle of contact surface were also used. All boxes were manufactured from heavy plywood by Guard Dog Products, UK, with standard dimensions to British Flyball Association guideline. In addition, contact surfaces were covered with a 5 mm rubberised, non-slip material (manufacturer unknown). All boxes used the same detachable ball release mechanism, which was inserted into each box prior to use.

A total of 11 Miqus M3 motion capture cameras (Qualysis, Göteborg, Sweden) were arranged on tripods at varying heights around the flyball box to capture a total volume of 5 m x 3 m x 2 m, allowing motion to be captured in 3 dimensions as each dog turned on the flyball box. Five cameras were positioned around the box, including one attached to a tripod with an extension mount to allow data from an overhead angle to be captured. The remaining cameras were positioned either side of the flyball run, to allow a total distance of 5 m of course to be captured. Lastly, an additional Miqus M3 camera was positioned facing the flyball box to capture standard 2-dimensional video. Once all cameras were in place and capture volume was confirmed the system was calibrated using the manufacturers standard, approved calibration method.

To enable the angles of joints of interest to be measured, reflective circular markers measuring 9.5 mm in diameter were attached to either side of each dog using a commercially available double-sided tape (3 M). All markers were placed on each animal by the same researcher to ensure location was consistent. The anatomical locations of interest as defined in previous studies^9,19,27^, were dorsal border of scapula spine, greater tubercle of the humerus, lateral epicondyle of the humerus, styloid process, lateral aspect metacarpal V, dorsal iliac spine, greater trochanter, lateral epicondyle of the femur, lateral malleolus and lateral aspect of metatarsal V. In addition, a marker was placed above the lumbosacral junction, as well as between each dogs’ shoulders at T3. Markers at the styloid process, lateral aspect metacarpal V, lateral malleolus and lateral aspect of metatarsal V were also secured using cohesive bandaging (Vetrap, 3 M, Bracknell, UK) to ensure they did not detach during high impact movement. The use of bandages does not interfere with the kinematics of the carpal joint in agility dogs ^20^, so was deemed suitable for the study.

Handlers prepared their dogs as they would prior to a flyball competitions, allowing acclimation to the research environment and equipment, and were directed by their usual handler throughout the study. Prior to data collection each dog was able to carry out a warmup in any manner their handler wished. Dogs were then encouraged to complete the course three times per box angle whilst turning to their usual side (left or right). Where dogs did not successfully retrieve the ball, they repeated the course until three successful ball fetches were completed. Once three successful runs were achieved the flyball box was replaced by one with a different angle and the process was repeated until all three angles had been used. The order in which the angles were used was randomised. To avoid fatigue, the maximum number of attempts a dog could have using any one angle was seven and dogs could also be withdrawn from the study by the owners at any time, however all dogs completed all three angles a minimum of three times successfully. All dogs were very experienced dogs and started the run at the same point, using always the same handler and box loader (person loading the ball on the box), to ensure they developed a similar speed and technique. Furthermore, a qualitative description of flyball turn, has described that there is a great intra-dog consistency, and therefore we were aware that dogs employ a very similar technique every time they complete the flyball turn.

### Kinematics data collection and analysis

Completion of each run was recorded at 300 fps using Qualisys motion capture software (QTM) version 2022.2 (Qualysis, Göteborg, Sweden) via a laptop connected to the camera array. Joints angles were measured on each video frame (300 frames per second) and included flexion and extension, as well as ROM of the hip, stifle and hock joints, shoulder, elbow and carpal joint during approach, take-off, box contact and landing from box, but in addition, maximum abduction of both the hip and shoulder joints was also collected during box contact. Also, additional analysis of the lowest phase of landing was conducted during the landing phase according to previous report^11^. The frame at which the dog had the minimum carpus angle during the time the first front foot touched the floor to the time the first rear foot hit the floor, was taken to be the lowest phase of the landing^28^. Minimum carpus angle, as well as shoulder angle at the lowest phase of the landing were then used for analysis. Duration of landing was measured in seconds (using video frames). The right forelimb was measured in the first instance with the left forelimb utilized if the angles of the right forelimb could not be clearly measured. Forelimb choice was consistent within the dog. All data was then collated using the manufacturers dedicated software.

### Statistical analysis

All statistical analysis were performed with SPSS (IBM Corp. Released 2021. IBM SPSS Statistics for Mac, Version 28.0. Armonk, NY: IBM Corp) and the confidence level was set as 95% (p < 0.05).

For the linear mixed effects model, data were divided into two subsets, one containing all parameters for forelimbs (FL) and another containing all parameters for the hindlimbs (HL). To analyse the prediction of parameter variance by the categorical descriptors of the obstacle, we reduced non-significant effects one-by-one, starting with the complete model until the final reduced model satisfied a minimal Akaike information criterion. First, backward random effects elimination was performed, followed by elimination of the fixed effects. P-values were calculated using Satterthwaite’s approximation for degrees of freedom.

The LMMs were used to investigate the main and interaction effects of ‘box angles’ (45, 60 and 83 degrees), the phase of ‘obstacle’ (approach, take-off, contact, push-off or landing) and the ‘limb’ (trail/lead or inner/outer) on joint angles considering the obstacles phases. For some joints angles, just the relevant obstacles phases were included, e.g. box push-off included only joints extension, or on contact phase only extension was analysed for carpal joint. The parameters ‘limb’, ‘obstacle’, and ‘box angle’ were included in the model as fixed effect. The full model considered the interactions obstacle*limb, obstacle*box angle, box angle*limb and box angle*limb*obstacle. ‘Dog’, which contained the individuals, was set as a random effect. One model was created for maximum flexion and extension of each joint. All models were fitted using restricted maximum likelihood estimation. Model acquirements and assumptions were fulfilled, as variances were homogeneous and residuals normally distributed. Post hoc analyses of main effects and interaction effects were carried out using comparison of least mean squares of group means with Bonferroni correction. The approximation for degrees of freedom is Satterthwaite’s. Significance was defined for *P* ≤ 0.05.

Despite being increasingly common in the analysis of biological data, LMM cannot provide analysis of variance to compare differences in kinematics variables between box angles at each phase of the obstacle (approach, TO, contact, push-off and landing) or between limbs at a specific box angle. Therefore, further tests were applied to analyse in more depth the changes in kinematics (Results of further tests can be seen in *the Supplementary material*). To compare the variables between flyball box angles, parametric data was analysed with one-way repeated measures analysis of variance (one-way repeated measures ANOVA), with distribution of variables as assessed by visual inspection of a boxplot and Greenhouse–Geisser correction applied when the sphericity was violated on the Mauchley’s test of sphericity. For the non-parametric data, Friedman’s test was used. Bonferroni correction for multiple pairwise comparisons was used, and the results report SPSS Bonferroni adjusted p-values (Supplementary *Tables S1-S4*). To compare lead and trail limbs or inner and outer limbs, data was consolidated and treated as related samples. As such paired t-test was used to compare inner and outer limbs when data was parametric, and Wilcoxon’s rank test was used when data was non-parametric (Supplementary *Tables S5 and S6*).

## Supplementary Information

Supplementary Video 1.

Supplementary Video 2.

Supplementary Information 1.

## Data Availability

The datasets generated during and/or analysed during the current study are available from the corresponding author on reasonable request.
