# Supplementary material for: 3D limb dynamics of flyball dogs turning on different box angles
Source: Sci Rep. 2024 Nov 13;14:27796. doi: 10.1038/s41598-024-78863-9 (PMC11561069; doi:10.1038/s41598-024-78863-9)
Supplement: Supplementary file 3 — Supplementary Information 1. [file 41598_2024_78863_MOESM3_ESM.docx]

**Supplementary Data**

Table S1 Flexion and extension of trail and lead joints of n=5 dogs during take-off towards three angulations of flyball boxes (45,50 and 83°) as well as associated statistical outputs, by repeated measures ANOVA or Friedman’s test. Data are stated as mean ± SD where parametric, and Median and IQR (Q1, Q3) where non-parametric. Superscript letters indicate a statistically significant difference (p<0.05).

| Variable | Joint | Flyball box angle | |  | Statistics outputs |
| --- | --- | --- | --- | --- | --- |
|  |  | 45° | 60° | 83° |  |
| Flexion (°) | Lead stifle | 76.24  IQR= 71.33, 88.67^a^ ⇑ | 107.16, IQR= 101.53, 115.83^b^ | 74.83, IQR= 59.20, 95.15^a^ ⇑ | χ2(2)=8.000, p =0.018 |
| Extension (°) | Trail hock | 114.07±25.51°^a^ ⇑ | 105.91±23.49^ab^ | 96.27±19.11°^b^ | F(2,12)=8.390, p=0.005 |
|  | Trail stifle | 81.21±14.18^a^ | 101.13±17.72  ^b^ ⇑ | 100.23±20.23^ab^ | F(2,14)=8.738, p=0.021 |
|  | Lead hip | 144.17±2.50^a^ ⇑ | 135.87±1.23^ab^ | 136.86±2.26^b^ | F(2,4)=8.251, p=0.038 |
|  | Trail elbow | 88.23±27.11^a^ | 102.44±21.78^ab^ | 123.34±30.23^b^ ⇑ | F(2,4)=12.122, p <0.001 |
|  | Lead shoulder | 142.87±3.80^a^ ⇑ | 122.344±2.88^b^ | 128.77±3.01^ab^ | F(1.195, 8.364)=9.340, p=0.003 |

Table S2 Flexion and extension of inner and outer limbs joints of n=5 dogs during contact/turn on three angulations of flyball boxes (45,50 and 83°) as well as associated statistical outputs, by repeated measures ANOVA or Friedman’s test.. Data are stated as mean ± SD where parametric, and Median and IQR (Q1, Q3) where non-parametric. Superscript letters indicate a statistically significant difference (p<0.05).

| Variable | Joint | Flyball box angle | |  | Statistics outputs |
| --- | --- | --- | --- | --- | --- |
|  |  | 45° | 60° | 83° |  |
| Flexion (°) | Outer hip | 95.69±13.77^a^⇑ | 111.13±15.67^ab^ | 112.34±8.72^b^ | F(2,4)=10.255, p=0.027 |
|  | Outer shoulder | 70.74±18.66^a^⇑ | 95.70°±20.28^b^ | 96.21±17.91^ab^ | F(2,14)=5.911, p=0.014 |
| Extension (°) | Inner hock | 122.56 IQR= 112.3, 132.71^ab^ | 123.56 IQR= 116.40°, 142.81^a^ | 137.54° IQR= 123.56^b^⇑ | X^2^(2)=6.00, *p*=0.050 |
|  | Outer carpus | 49.64±6.82^a^ | 74.11±3.83^b^ | 78.07±4.04^c^⇑ | F(2,7)=11.122, p<0.13 |
| Abduction (°) | Hip | 101.23 IQR= 98.67, 107.06^a^⇑ | 93.88 IQR= 86.49, 99.30^b^ | 89.60 IQR= 76.41, 98.99^b^ | χ2(2)=7.714, p=0.021 |

Table S3 Flexion and extension of inner and outer limbs joints of n=5 dogs during push off on three angulations of flyball boxes (45,50 and 83°) as well as associated statistical outputs, by repeated measures ANOVA or Friedman’s test.. Data are stated as mean ± SD where parametric, and Median and IQR (Q1, Q3) where non-parametric. Superscript letters indicate a statistically significant difference (p<0.05).

| Variable | Joint | Flyball box angle | |  | Statistics outputs |
| --- | --- | --- | --- | --- | --- |
|  |  | 45° | 60° | 83° |  |
| Extension (°) | Outer hock | 132.81 IQR= 129.72, 142.15^a^ | 157.86 IQR= 152.35, 171.30^b^⇑ | 155.30 IQR= 152.52, 170.20^b^⇑ | χ2(2)=6.750, p=0.034 |
|  | Inner stifle | 119.67 IQR= 114.45, 120.22^a^ | 129.79 IQR= 122.28, 149.52^b^⇑ | 140.39 IQR= 134.74, 153.19^b^⇑ | χ2(2)=9.250, p=0.010 |

Table S4 Flexion and extension of inner and outer limbs joints of n=5 dogs during landing from three angulations of flyball boxes (45,50 and 83°) as well as associated statistical outputs, by repeated measures ANOVA or Friedman’s test.. Data are stated as mean ± SD where parametric, and Median and IQR (Q1, Q3) where non-parametric. Superscript letters indicate a statistically significant difference (p<0.05).

| Variable | Joint | Flyball box angle | |  | Statistics outputs |
| --- | --- | --- | --- | --- | --- |
|  |  | 45° | 60° | 83° |  |
| Flexion (°) | Lead Shoulder | 72.86±2.45^a^⇑ | 91.41±3.94^a^⇑ | 104.45±5.34^b^ | F(2,14)=17.193, p<0.001 |
|  | Trail Elbow | 85.78 IQR= 56.38,77.39^a^ | 50.64 IQR= 39.48, 69.00^b^⇑ | 72.71 IQR=44.60, 87.15^ab^ | χ^2^(2)=7.161, p =0.028 |

Table S5 Flexion and extension of inner and outer limbs joints of n=5 dogs for all box angles during contact/turn and push off as well as associated statistical outputs, by paired t-test or Wilcoxon’s test. Data are stated as mean ± SD where parametric, and Median and IQR (Q1, Q3) where non-parametric. Superscript letters indicate a statistically significant difference (p<0.05).

| Variable | Phase of obstacle | Joint | Limb | | Statistics Outputs |
| --- | --- | --- | --- | --- | --- |
|  |  |  | Inner | Outer |  |
| Flexion (°) | Contact/turn | Stifle | 82.25 IQR= 73.26, 101.31^a^ | 75.15 IQR= 65.23, 92.44^b^ ⇑ | *z*=2.452, p=0.014 |
|  |  | Shoulder | 94.68° IQR= 83.98°, 110.99^a^ | 91.23° IQR= 69.27, 104.94^b^⇑ | z=-2.184, p=0.029 |
| Extension (°) | Contact/turn | Stifle | 147.04 IQR= 127.89, 166.66⇑ | 131.95 IQR= 116.73, 160.71 | *z*=3.086, p=0.014 |
|  |  | Hip | 124.96 IQR= 112.16, 136.14^a^ | 145.61 IQR= 137.37 155.70^b^⇑ | *z*=3.425, p<0.001 |
|  |  | Carpus | 77.38 IQR= 67.22, 86.19^a^⇑ | 72.69 IQR= 64.84, 77.77^b^ | *z* = -2.954, p=0.003 |
|  |  | Shoulder | 140.87 IQR= 133.72, 157.80^a^⇑ | 132.74 IQR= 130.37, 136.26^b^ | z =-4.126, p<0.001 |
|  | Push off | Hock | 148.80 IQR= 133.36, 167.18^a^ | 152.47 IQR= 136.56,167.66^b^⇑ | z=2.376, p=0.017 |
|  |  | Stifle | 135.49±16.76^a^⇑ | 128.07±16.12^b^ | t(36)=2.173, p =0.036 |
|  |  | Hip | 124.80 IQR= 118.47, 135.34^a^ | 142.64 IQR= 130.18, 153.38^b^⇑ | *z*=3.877, p<0.001 |

Table S6 Flexion and extension of trail and lead limbs joints of n=5 dogs for all box angles during contact/turn and push off as well as associated statistical outputs, by paired t-test or Wilcoxon’s test. Data are stated as mean ± SD where parametric, and Median and IQR (Q1, Q3) where non-parametric. Superscript letters indicate a statistically significant difference (p<0.05).

| Variable | Phase of obstacle | Joint | Limb | | Statistics Outputs |
| --- | --- | --- | --- | --- | --- |
|  |  |  | Trail | Lead |  |
| Flexion (°) | Landing | Shoulder | 121.51 IQR= 115.42, 141.66^a^ | 110.23 IQR= 101.23, 120.42^b^⇑ | *z*=-2.666, p=0.008 |
| Extension (°) | Landing | Carpus | 48.79±0.707^a^⇑ | 40.03±1.81^b^ | t(36)= 4.404, p=0.004 |

Table S7. Demographic data of dogs participating in 3D data capture

| Dog | Age (years) | Weight (Kg) | Wither height (cm) | Direction of turn |
| --- | --- | --- | --- | --- |
| 1 | 6 | 12 | 43 | Left |
| 2 | 2 | 9 | 38 | Left |
| 3 | 8 | 18 | 48 | Left |
| 4 | 6 | 12 | 40 | Right |
| 5 | 5 | 14.5 | 35 | Right |
